# Acute Plastic Deformation of the Forearm Involving Both Bones in an Adolescent: Distinct Deformity Vectors Requiring Individualized Three-Dimensional Correction

**DOI:** 10.7759/cureus.109679

**Published:** 2026-05-26

**Authors:** Atsuya Kameda, Taku Hatta, Atsushi Kogure, Mika Abe, Gaku Matsuzawa

**Affiliations:** 1 Orthopedic Surgery, Joint Surgery, Sports Clinic Ishinomaki, Ishinomaki, JPN; 2 Orthopedic Surgery, Japanese Red Cross Ishinomaki Hospital, Ishinomaki, JPN; 3 Orthopedic Surgery, Sendai City Hospital, Sendai, JPN; 4 Orthopedic Surgery, Tome Citizen Hospital, Tome, JPN

**Keywords:** acute plastic deformation, corrective osteotomy, forearm, radius, ulna

## Abstract

Acute plastic deformation of both the radius and ulna without fracture is rare in adolescents and can be difficult to recognize from initial examination or radiologic assessment. A 16-year-old female sustained a bicycle-related injury and subsequently developed progressive limitation of forearm rotation, prompting reevaluation. Preoperative forearm rotation was restricted to 15 degrees of supination and 60 degrees of pronation, indicating substantial functional impairment. Three-dimensional deformity analysis revealed distinct maximal inclination directions for the radius and ulna, allowing determination of optimal correction angles from the contralateral side. A step-cut osteotomy for the radius and a hinged osteotomy for the ulna achieved adequate correction, supported by intraoperative rotational assessment. At two months postoperatively, forearm rotation had improved to 100 degrees of supination and 90 degrees of pronation, enabling full return to badminton at three months. This case highlights the diagnostic difficulty of acute plastic deformation involving both the radius and ulna within a single forearm and underscores the importance of precise three-dimensional planning in achieving successful correction.

## Introduction

Acute plastic deformation (APD) of the forearm is an uncommon injury that predominantly occurs in children and young adolescents, whose bones possess greater elasticity than those of adults [1]. In this age group, bone can undergo plastic, irreparable deformation when the applied force exceeds the elastic limit but remains insufficient to cause a cortical break, resulting in bowing without a visible fracture line [2]. Therefore, the diagnosis of APD is often challenging, and delayed recognition has been reported in both pediatric and adult populations [3]. Missed or late diagnosis may result in persistent pain, restricted forearm rotation, and the need for corrective osteotomy.

Most cases of APD involve isolated bowing of either the radius or the ulna, typically following low-energy trauma [2,4]. In contrast, APD affecting both forearm bones without associated fractures or dislocations is exceedingly rare. Only a few cases have been documented in the literature, and the optimal management strategy remains unclear [5,6]. Accurate assessment of deformity, careful evaluation of distal radioulnar joint (DRUJ) stability, and appropriate timing of intervention are essential for achieving satisfactory outcomes. Because forearm rotation depends on the radius rotating around a relatively fixed ulna, even subtle deformities of either bone can lead to clinically significant limitations in pronation and supination [7].

Corrective osteotomy is indicated when functional impairment with significant deformity is identified. However, achieving precise correction can be technically demanding, especially when deformities occur in multiple planes. Preoperative planning based on three-dimensional assessment and intraoperative methods to confirm rotational alignment may improve surgical accuracy, yet reports describing such strategies in APD are limited.

We present a rare case of APD of the forearm involving both the radius and ulna in a young athlete, managed with detailed preoperative planning and corrective osteotomies of both bones. A step-cut osteotomy for the radius and a hinged osteotomy for the ulna were performed, and an external fixator was used intraoperatively as a dynamic reduction aid to confirm rotational alignment. This report highlights the diagnostic challenges of APD without fracture and introduces a practical intraoperative technique to enhance the accuracy of deformity correction.

## Case presentation

A 16-year-old right-handed female high school student with a lean body habitus (height 160 cm, weight 40 kg) and membership in a school badminton club sustained an injury after falling from a bicycle and being run over by another bicycle. She noted pain in the forearm and wrist but had no visible deformity. She was evaluated at a local emergency clinic on the day of injury, where only wrist radiographs were obtained despite her reporting forearm pain, and no fracture was identified. She was treated with topical medication and referred to our institution. Written informed consent for the publication of this case and its accompanying images was obtained from the patient.

At her first visit the following day, she reported wrist pain, particularly during forearm supination. Physical examination revealed mild tenderness around the distal radioulnar joint (DRUJ) without swelling or ecchymosis. The ballottement test was negative. Forearm radiographs were again not obtained at this visit, and only wrist radiographs were taken, which showed no obvious abnormalities. She was diagnosed with a wrist sprain and treated conservatively.

One week later, she returned with mild residual pain during gripping but no motion pain in the wrist. Four weeks after the initial visit, she presented again with persistent ulnar-sided wrist pain. Examination revealed DRUJ tenderness and a now-positive ballottement test. Range of motion showed elbow flexion to 150 degrees and extension to 10 degrees of hyperextension. Forearm rotation was markedly limited, with 15 degrees of supination and 60 degrees of pronation. Wrist motion was also restricted, with 50 degrees of palmar flexion and 60 degrees of dorsal flexion.

Forearm radiographs demonstrated bowing deformities of both the radius and ulna. The radius showed radial-dorsal convexity, and the ulna showed volar convexity, consistent with APD of both forearm bones without fracture (Figure 1). Because of persistent ulnar-sided wrist pain and DRUJ tenderness, an MRI of the wrist was obtained at four weeks after the injury to evaluate for a possible triangular fibrocartilage complex (TFCC) injury. MRI revealed dorsal subluxation of the DRUJ without interposed soft tissue (Figure 2). A CT scan was subsequently performed at five weeks after the injury to facilitate three-dimensional preoperative planning for corrective osteotomy.

**Figure 1:**
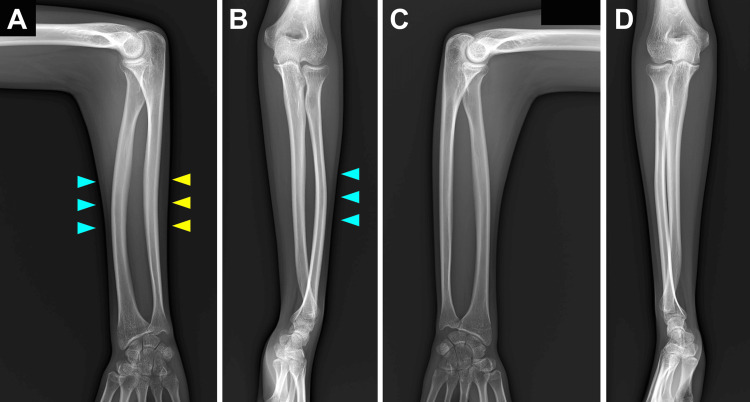
Preoperative radiographs of both forearms. Radiographs of the right forearm (A, B) showing radial–dorsal bowing of the radius (blue arrowheads) and volar bowing of the ulna (yellow arrowheads). Corresponding radiographs (C, D) of the left forearm. No fractures are visible in either forearm.

**Figure 2:**
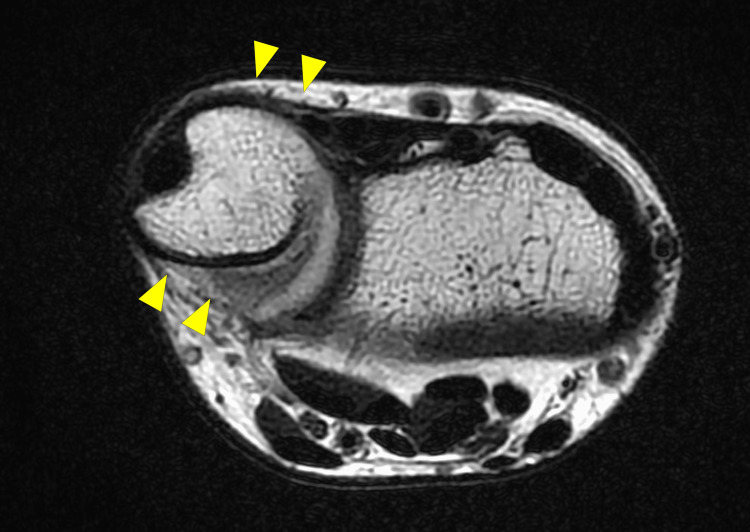
Preoperative MRI of the wrist. The axial T2-weighted image on MRI demonstrating dorsal subluxation of the distal radioulnar joint (DRUJ) without interposed soft tissue (yellow arrowheads).

Corrective osteotomy was planned using the three-dimensional Image Analysis System (Synapse Vincent, Fujifilm Medical, Tokyo, Japan) to obtain precise three-dimensional measurements of the deformities in both the radius and ulna from CT. Because the deformity patterns differed between the two bones, separate anatomical reference joints were used for analysis.

For the radius, deformity measurements were obtained using the wrist joint as the reference. Comparison with the contralateral side demonstrated 3.9 degrees of ulnar deviation and 3.9 degrees of volar angulation. Vector analysis showed that the maximal inclination was directed 45 degrees toward the volar-ulnar quadrant. When visualized in the coronal plane with the radial side positioned at the twelve o’clock orientation, the deformity pointed toward the one-thirty position, indicating a combined volar and ulnar direction. The optimal inclination angle for correction was determined from the uninjured side and measured 11.9 degrees. Based on these findings, a step-cut osteotomy was selected to maximize bony contact and provide stable fixation (Figure 3).

**Figure 3:**
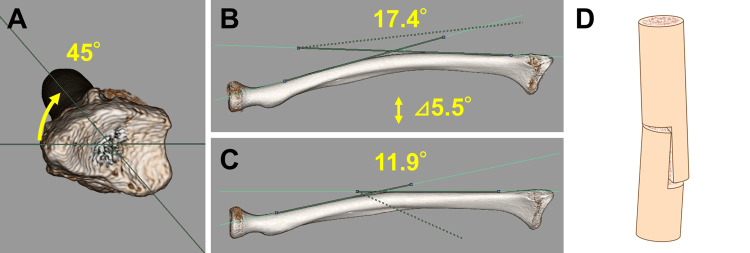
Three-dimensional deformity analysis of the radius. Three-dimensional CT reconstruction images illustrate the deformity. All deformity measurements were performed using Synapse Vincent. An image viewed from the wrist joint surface (A) demonstrates the maximal inclination directed 45 degrees toward the volar–ulnar quadrant. A full-length 3D reconstruction obtained at the rotational angle that best visualizes the deformity direction (B) and a mirrored reconstruction of the contralateral radius at the corresponding angle (C) show an altered inclination angle of 5.5 degrees. A schematic illustration (D), created by the authors using Microsoft PowerPoint, depicts the planned step-cut osteotomy based on the measured deformity direction and inclination angle.

For the ulna, deformity measurements were obtained using the elbow joint as the reference. Comparison with the contralateral side revealed 1.8 degrees of radial deviation and 12.4 degrees of dorsal angulation. Vector analysis demonstrated that the maximal inclination was oriented 8.3 degrees toward the radial side from the dorsal aspect. When viewed in the coronal plane with the radial side at the twelve o’clock orientation, the deformity direction corresponded to slightly past the nine o’clock position, indicating a dorsal-to-radial direction. The optimal inclination angle, calculated from the uninjured side, was 5.4 degrees. A hinged osteotomy preserving the volar cortex was planned to maintain stability and promote bone healing (Figure 4).

**Figure 4:**
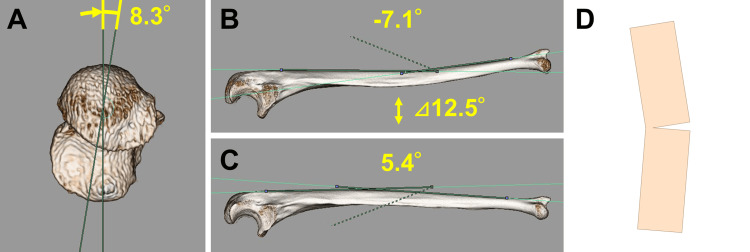
Three-dimensional deformity analysis of the ulna. Three-dimensional CT reconstruction images illustrate the ulnar deformity. All deformity measurements were performed using Synapse Vincent. An image viewed from the elbow joint surface (A) demonstrates the maximal inclination directed 8.3 degrees toward the radial side from the dorsal aspect. A full-length 3D reconstruction obtained at the rotational angle that best visualizes the deformity direction (B) and a mirrored reconstruction of the contralateral ulna at the corresponding angle (C) show an altered inclination angle of 12.5 degrees. A schematic illustration (D), created by the authors using Microsoft PowerPoint, depicts the planned hinged osteotomy preserving the volar cortex, based on the measured deformity direction and inclination angle.

Surgery was performed six weeks after injury. The radius was exposed through a dorsal approach, and a step-cut osteotomy of the radius was created at a 45-degree radial-dorsal inclination relative to the wrist joint surface, and fixation was achieved using a Small 3.5‑mm locking compression plate (LCP) (DePuy Synthes, Warsaw, IN, USA; 5‑hole) pre-bent to 12 degrees, providing approximately 6 degrees of correction. The ulna was approached through a medial subcutaneous incision, and a hinged osteotomy of the ulna was performed at an 8-degree radial-dorsal inclination relative to the elbow joint surface, and fixation was achieved with a Small 3.5‑mm LCP (DePuy Synthes; 6‑hole) pre-bent to 5 degrees, providing approximately 13 degrees of correction.

Before final fixation of the ulna, an external fixator was temporarily applied to assess intraoperative forearm rotation dynamically. A Hoffmann II external fixator (Stryker, Kalamazoo, MI, USA) was used for this temporary assessment. This allowed confirmation of improved rotational alignment and facilitated accurate plate placement. Care was taken to avoid disrupting the hinge during correction (Figure 5).

**Figure 5:**
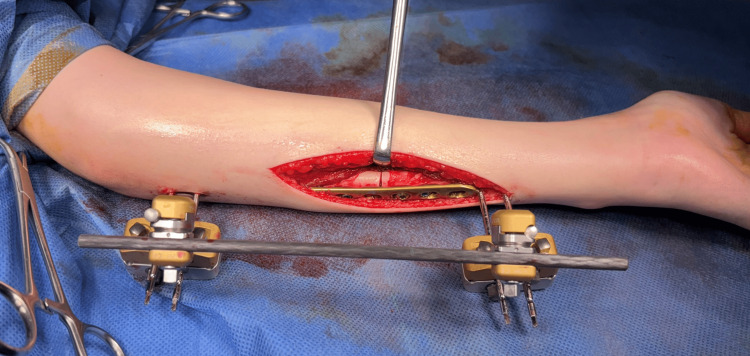
Intraoperative assessment of forearm rotation. An intraoperative photograph showing the use of a temporary external fixator to evaluate forearm rotation before final fixation of the ulna with a locking plate.

Postoperative management consisted of immobilization in a Muenster cast for two weeks, followed by gradual mobilization. Light activities such as bicycle riding were permitted at six weeks postoperatively. At two months, the patient demonstrated substantial improvement in range of motion, with elbow flexion of 150 degrees and extension to 0 degrees, forearm rotation of 100 degrees in supination and 90 degrees in pronation, and wrist motion of 75 degrees in palmar flexion and 80 degrees in dorsiflexion. The patient resumed badminton activities at three months postoperatively without limitations. One year after the surgery, she remains asymptomatic, and the range of motion has further improved to 150 degrees of elbow flexion, 0 degrees of extension, 100 degrees of supination, 90 degrees of pronation, 85 degrees of palmar flexion, and 90 degrees of dorsal flexion (Figure 6).

**Figure 6:**
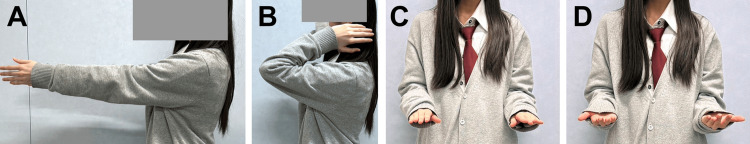
Postoperative range of motion at one year. Elbow flexion (A) and extension (B), and forearm pronation (C) and supination (D) at one year postoperatively.

Radiologically, complete bone union without any residual deformity was confirmed at both radius and ulna at six months postoperatively (Figure 7).

**Figure 7:**
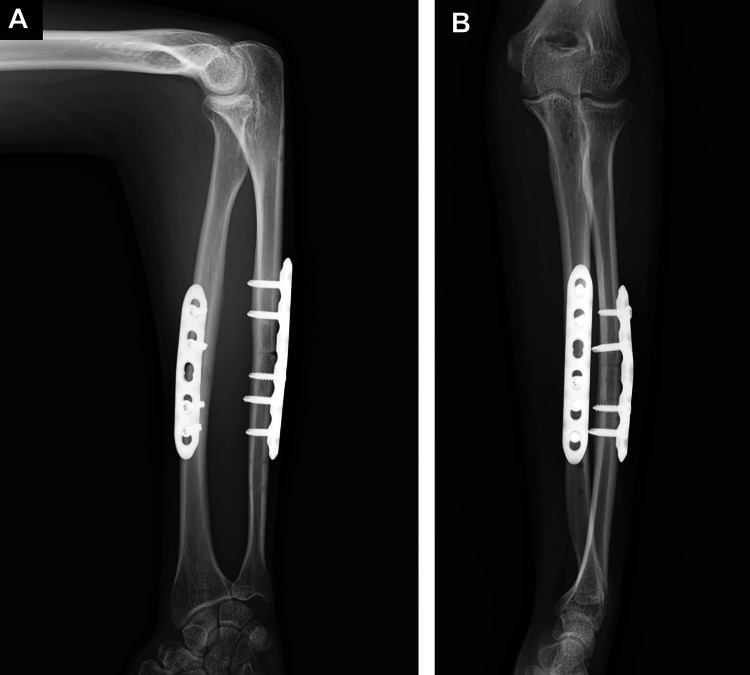
Radiographs at six months postoperatively. Postoperative radiographs (A, B) showing complete bone union of both the radius and ulna without residual deformity.

## Discussion

APD of the forearm is an uncommon injury, particularly in adolescents, and its diagnosis is often challenging because no fracture line is visible on radiographs [1]. Delayed recognition may lead to persistent pain, restricted forearm rotation, and the need for corrective osteotomy [2]. Most reported cases involve isolated bowing of either the radius or the ulna, whereas simultaneous deformity of both bones without associated fractures is exceedingly rare [6]. This case highlights the diagnostic difficulty of APD involving both forearm bones and underscores the importance of careful clinical assessment when symptoms persist despite unremarkable initial imaging.

A notable diagnostic pitfall in this case was that forearm radiographs were not obtained at the initial evaluations, even though the patient reported forearm pain. Because APD often presents without visible deformity and with subtle symptoms, limiting imaging to the wrist can lead to a missed diagnosis. When APD is suspected, particularly in adolescents with persistent pain or restricted rotation, dedicated forearm radiographs in two planes should be obtained, and further evaluation with CT may be warranted to assess subtle bowing deformities. This clinical course illustrates how APD can be overlooked when deformity is subtle and emphasizes the need for a high index of suspicion in young patients whose bone elasticity predisposes them to plastic deformation without overt fracture.

Precise preoperative planning is essential for achieving appropriate correction in APD, especially when deformities occur in multiple planes [2]. In this case, three-dimensional analysis from preoperative CT images enabled detailed deformity of each bone. Importantly, the maximal inclination directions differed between the radius and ulna, necessitating individualized planning. For the radius, the maximal deformity was directed 45 degrees toward the volar-ulnar quadrant when referenced to the wrist joint, corresponding to the one-thirty position in the coronal plane. In contrast, the ulna demonstrated maximal inclination of 8.3 degrees toward the radial side from the dorsal aspect when referenced to the elbow joint, corresponding to slightly past the nine o’clock position. These directional differences highlight the complexity of the deformities and the value of vector-based analysis in determining the optimal correction angle derived from the contralateral uninjured side for both bones.

The surgical strategy was tailored to the characteristics of each deformity. A step-cut osteotomy was selected for the radius to maximize bony contact and provide stable fixation, whereas a hinged osteotomy preserving the volar cortex was chosen for the ulna to maintain stability during correction. We adopted temporary application of an external fixator, since a previous study demonstrated this technique allows dynamic intraoperative assessment of forearm rotation, facilitating accurate alignment before final fixation [6]. Although the referenced technique was originally described in adult patients with plastic deformation, the fundamental concept of using an external fixator to assess rotational alignment is applicable across age groups. In the present case, the device was applied only temporarily as an intraoperative alignment guide without applying corrective force, which we considered appropriate and safe for an adolescent patient with ongoing skeletal development. This technique is particularly useful when rotational deformity is difficult to judge under direct visualization alone.

The postoperative course was favorable. After two weeks of immobilization, gradual mobilization was initiated, and light activities such as bicycle riding were permitted at six weeks. Range of motion improved steadily, with near-complete restoration of elbow, forearm, and wrist motion by two months and further gains at one year postoperatively. The patient returned to badminton at three months without limitations. These outcomes suggest that adequate deformity correction, combined with appropriate postoperative rehabilitation, can lead to excellent functional recovery even in complex cases of APD involving both the radius and ulna.

This case demonstrates that APD without fracture can present subtly and may be overlooked on initial evaluation. Three-dimensional deformity analysis using digital planning software, combined with individualized osteotomy techniques, can facilitate precise correction and favorable clinical outcomes. Awareness of this injury pattern and the use of advanced planning tools may help prevent delayed diagnosis and improve treatment results in similar cases.

## Conclusions

This case highlights the diagnostic difficulty of APD involving both the radius and ulna in adolescents. Three-dimensional deformity analysis was essential for identifying the distinct maximal inclination directions of each bone and for determining the optimal correction angles based on the uninjured side. Individualized osteotomy techniques tailored to each deformity allowed adequate correction and resulted in excellent functional recovery. Awareness of this injury pattern and the use of precise preoperative planning tools may help prevent delayed diagnosis and improve outcomes in similar cases.
